# Supplementary figures and images for: SARS-CoV-2 infection enhancement by amphotericin B: implications for disease management
Source: J Virol. 2025 Jun 4;99(7):e00519-25. doi: 10.1128/jvi.00519-25 (PMC12282131; doi:10.1128/jvi.00519-25)

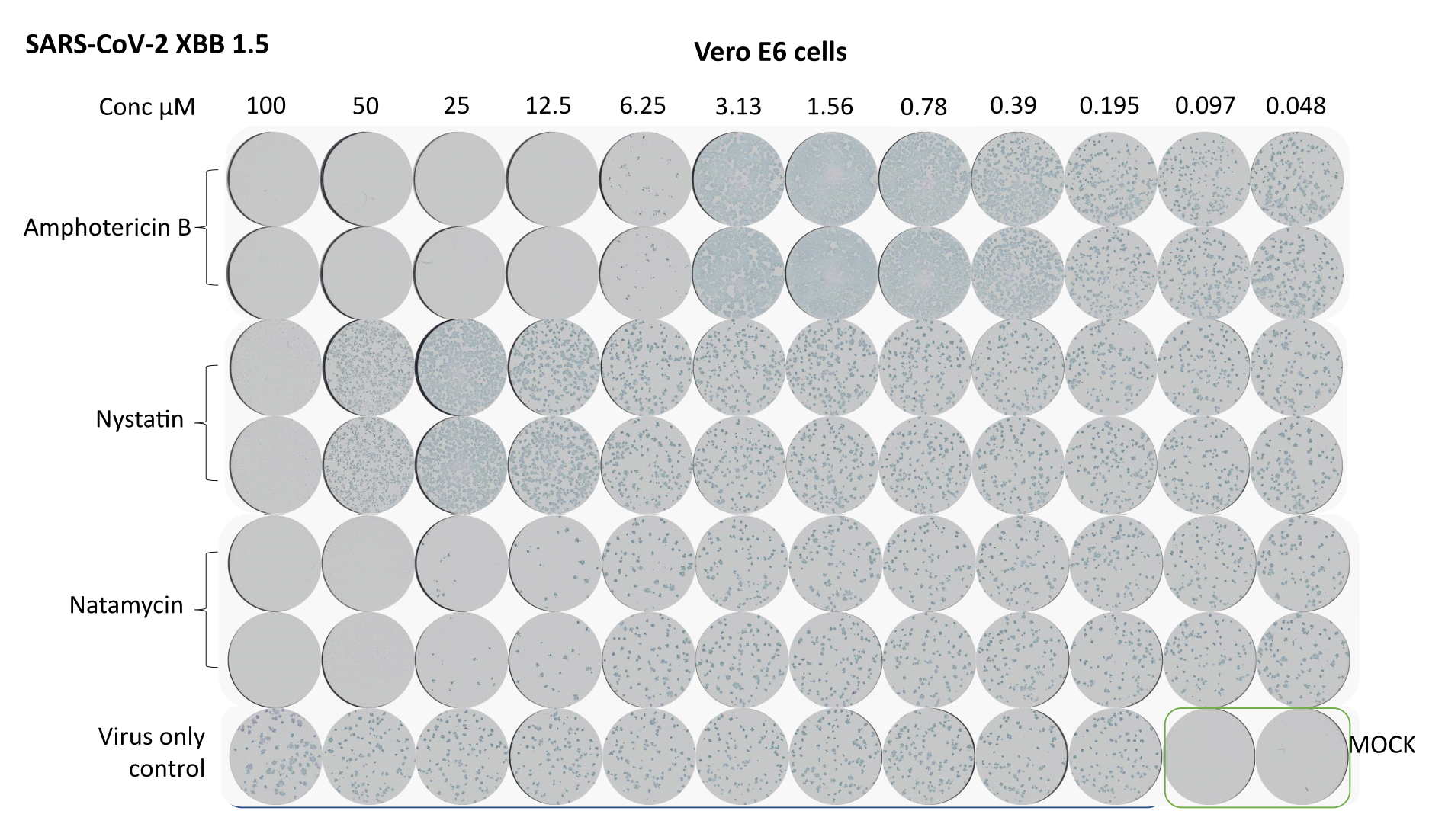

Supplement: Figure S1 — Effects of antifungals on SARS-CoV-2 XBB 1.5. [file jvi.00519-25-s0001.tiff]
